# Exo-Tox: Identifying Exotoxins from secreted bacterial proteins

**DOI:** 10.1186/s13040-025-00469-2

**Published:** 2025-08-08

**Authors:** Tanja Krueger, Damla A. Durmaz, Luisa F. Jimenez-Soto

**Affiliations:** 1 Walther-Straub Institute of Pharmacology and Toxicology, Ludwig-Maximilians-Universität in Munich, Goethestrasse, 80336 Munich, Bavaria Germany; 2 Department of Informatics, Unit for Bioinformatics and Computational Biology, Technical University of Munich School of Computation, Information and Technology, Boltzmannstrasse, 85748 Garching/Munich, Bavaria Germany

**Keywords:** Bacterial, Toxins, Exotoxins, Proteins, Predictor, Embeddings

## Abstract

**Background:**

Bacterial exotoxins are secreted proteins able to affect target cells, and associated with diseases. Their accurate identification can enhance drug discovery and ensure the safety of bacteria-based medical applications. However, current toxin predictors prioritize broad coverage by mixing toxins from multiple biological kingdoms and diverse control sets. This general approach has proven sub-optimal for identifying niche toxins, such as bacterial exotoxins. Recent Protein Language Models offer an opportunity to improve toxin prediction by capturing global sequence context and biochemical properties from protein sequences.

**Results:**

We introduce Exo-Tox, a specialized predictor trained exclusively on curated datasets of bacterial exotoxins and secreted non-toxic bacterial proteins, represented as embeddings by Protein Language Models. Compared to Basic Local Alignment Search Tool (BLAST)-based methods and generalized toxin predictors, Exo-Tox outperforms across multiple metrics, achieving a Matthews correlation coefficient > 0.9. Notably, Exo-Tox’s performance remains robust regardless of protein length or the presence of signal peptides. We analyze its limited transferability to bacteriophage proteins and non-secreted proteins.

**Conclusion:**

Exo-Tox reliably identifies bacterial exotoxins, filling a niche overlooked by generalized predictors. Our findings highlight the importance of domain-specific training data and emphasize that specialized predictors are necessary for accurate classification. We provide open access to the model, training data, and usage guidelines via the LMU Munich Open Data repository.

## Introduction

Bacterial toxins include the most potent toxins known today. Many have been directly linked to deadly human diseases, such as botulinum toxin, cholera toxin, or diphteria toxin. It has been hypothesized that bacteria produce them for defense (e.g. evasion of predators) or to allow the colonization of preoccupied habitats [1–3]. Whatever their objective is, their origins are diverse, and many are known to originate from horizontal gene transfer [4, 5] through bacteriophages.

When considering bacterial toxins, it is necessary to distinguish between endotoxins and exotoxins. The first are of lipidic nature and released upon physical disruption of bacterial membranes. The second, exotoxins, are actively secreted proteins. Their secretion occurs either into the extracellular space or directly into the target cells involving diverse sets of pathways and secretion signals [6–8]. Our focus is on bacterial exotoxins, which we define as: secreted proteins capable of cellular manipulation of a target organism.

With the identification of bacterial toxins, specifically diphteria toxin in the 1800 s [9], a new understanding of human diseases caused by bacteria started, leading to the development of a vaccine against diphteria, even though the connection had not always been immediately understood.

With emerging bacteria based treatments like probiotics [10–12] or fecal microbiota transplantation [13, 14], any method able to predict bacterial exotoxins from protein sequences will improve their safety. Identification of exotoxins may also lead to the discovery of novel drug candidates, considering that some exotoxins have been proven to be suitable pharmaceutical agents [15–17].

Traditional wet-lab approaches like genomewide mutagenesis have helped to identify toxins and their importance in infection for bacteria growing in the lab. For larger scale, this approach is not economically feasible or possible. In-silico methods using the amino acid sequence of proteins are a promising alternative. Several of these approaches include techniques based on sequence similarity analyzing and clustering tools, (e.g.BLAST [18], PSI-BLAST [19]; or MMseqs2 [20]), and homology methods based on Hidden Markov Models(HMMs) [21] such as HMMER [22]. These, however, are often limited to implicit (HMMs) or explicit (BLAST) local sequence motives to identify toxins [23].

Efforts to predict toxins using Machine Learning vary in their focus and approach. Many models are designed specifically for one toxin origin [24–27], peptides [28–33], specifically for bacteriocins [34–36] or only effector proteins [37, 38].

By contrast, other predictors [39–45] expand their scope to include a broader set of virulence factors, or proteins associated with pathogenicity, and are not limited to classical toxins.

Recently, generalized toxin predictors have emerged combining toxins from multiple biological origins, including animals, plants, and bacteria [28, 46–49]. These predictors often included very diverse non-toxin control sets, including proteins from different origin species than the toxins, or proteins with sub-cellular localizations distinct from secreted toxins [26, 46, 47].

Toxins predictors also differ in their input features and architecture. The most recent, CSM-Toxin [47], uses a modification of ProteinBERT [50], a protein language Model (pLM), and aims to find any type of toxin independent of origin. pLMs are pre-trained models that learn contextual relationships between amino acids in a protein sequence, analogous to how language models capture the meaning of words within a sentence. Once trained, a pLM encodes each protein sequence into a numerical vector known as an embedding. These embeddings serve as a compact, information-rich representation of the protein and can be used as input for specialized downstream predictors, including toxin classifiers.

Importantly, these embeddings have been shown to outperform traditional protein representations such as amino acid k-mers or structure-derived features across a wide range of predictive tasks [51]. Studies have demonstrated that pLM embeddings capture a broad spectrum of biologically relevant properties, from basic features like sequence length and water solubility, to complex information such as secondary and tertiary structure, protein fold, intrinsic disorder, subcellular localization, and even evolutionary information [52–55].

Taking advantage of the T5 architectures used for the pLM model ProtT5 [54], and realizing the lack of a bacterial exotoxin predictor trained in only bacterial exotoxins, we present here Exo-Tox: a specialized predictor that closes the gap of identifying bacterial exotoxins from secreted non-toxic bacterial proteins using the protein’s primary sequence and protein Language Models (pLMs). Our model uses a highly curated dataset of bacterial exotoxins, defined here as secreted proteins capable of cellular manipulation of a target organism. As negative label, non-toxic secreted bacterial proteins were used. Exo-Tox is the name given to our best performing model, after evaluation of two predictors with different sets of input features: a naive approach of amino acid composition (*aac*), and protein embeddings generated by protT5 (Embs20). Exo-Tox identifies the toxin potential of secreted bacterial proteins more reliably than a generalized toxin prediction tool, a BLAST approach, CSM-Toxin predictor, and MultiToxPred 1.0. When compared with similarity based on structural predictions (Foldseek), Exo-Tox was comparable. We investigated the relevance of signal peptides, and protein length in their performance. None of them play a role in the capacity of the predictor to classify toxins. To evaluate the applicability and transferability of the Exo-Tox, we applied it to two related proteins sets, bacteriophages (natural transmitters of toxins) and general bacterial proteins, and we present these results.

## Methods

### General information

We built a predictor to differentiate bacterial exotoxins and secreted bacterial non-toxin. We compared two input feature approaches to a sequence similarity based method. Input features included the amino acid composition and an embedding based feature set. For the selected approach we investigated the biological relevance and robustness twofold: by testing partial sequences without the signal peptides and by retraining on scrambled sequences.

### Data accessibility

All code used for data analysis is accessible in the LMU University repository https://doi.org/10.5282/ubm/data.576. As stated in the data availability statement, raw data and code for data wrangling that support the findings of this study are openly available in LMU repository. For the review process, a new set of raw data, predictor and analysis were added resulting in this manuscript’s data. The data for the review process can be found under https://doi.org/10.5282/ubm/data.665.

### Raw data

The raw data consists of a set of expert curated bacterial exotoxins and a set of secreted, bacterial non-toxins previously published https://doi.org/10.5282/ubm/data.423 and analyzed under [56]. The dataset contains exotoxins from four bacterial toxin types, its sequences downloaded from Swiss-Prot (UniProtKB/Swiss-Prot (RRID:SCR_021164)) [57] and PubMed (PubMed (RRID:SCR_004846)) [58]. The database includes only active subunits of toxins. All sequence labeled as “fragment” or “partial” were removed. We also excluded any other virulence factors, and protein-based toxins that were not produced by bacteria. The total number of exotoxins is 2396 sequences. The non-toxins set is based on the PSORTb 3.0b, a database containing the predicted sub-cellular localization of bacterial protein [59]. Sequences with predicted association to membranes, outer membrane vesicles (OMVs), periplasm, or cytoplasm were removed, leaving only secreted proteins(9082 sequences). The full description of the data curation is described in Kruger et al, 2024 [56].

### Redundancy reduction

Raw datasets may contain sequences that are highly similar or identical to each other, as a result from biological processes (e.g horizontal gene transfer), or from artificial sources (e.g. sampling bias during data collection, labeling). To avoid overestimating the predictive power of our models by including redundant sequences, we performed two steps of redundancy reduction using the MMseqs2 algorithm [20]. First, we removed duplicates between toxins and non-toxins with MMseqs2 using the easy-cluster option and its default parameters. We manually set the similarity threshold of 100% and the alignment coverage mode to 0, which causes that both the query and the target sequences must be fully covered by the alignment at the specified threshold. We retained only the representative sequence from each cluster, and removed any sequences that were present in both the toxin and non-toxin sets from the non-toxin control proteins. Second, we reduced the sequence similarity within the toxins and non-toxins datasets independently with a similarity threshold of 30%. This step was carried out before the separation of the test set and the cross validation folds. This approach makes sure that no two proteins between the sets shared more than the selected 30% sequence similarity. The resulting redundancy-reduced datasets contain 1069 toxins and 1308 non-toxins.

### Generating the test set

We generated a hold-out test set, through stratified splitting, with the label as stratifying factor and a split ratio of 85% training/validation and 15% test sequences. The selection of the test set did not use other datasets (benchmarks) because of their inclusion of proteins that were non-toxins but virulence associated, or from different kingdoms. Considering we reuse this test-set to compare our work with the existing predictor CSM-Toxin, our final test set was equally redundancy reduced against our own- and the CSM-Toxin training set. For this we used MMseqs2 with a similarity threshold of 30% and modus 0. Any subsequent steps of scaling, or features selection was performed independently on the training and validation set, without taking the test set into account.

### Vector representation of protein sequences - input features

#### Generation of vectors of amino acid composition

We tried different protein representations as input features. For the naive approach, we used the amino acid composition (*aac*) of proteins calculated with Eq. 1. The resulting feature vector contains 20 individual values, which were scaled as described under “Prediction method” section.

1 $$\begin{aligned} {Ratio(i)}= \frac{n(i)}{ n(all\, amino\, acids) } \end{aligned}$$

where:

$$\begin{aligned} i & = \text {specific amino acid}\\ n & = \text {number of occurrences} \end{aligned}$$

#### Generation of embeddings based input features

To obtain the embedding representation of each protein, we used the pre-trained Protein Language de(pLM) ProtT5 [54] version ProtT5-XL-UniRef50 (also referred to as ProtT5-XL-U50). We used their embed_ProtT5.ipynb colab notebook to translate the protein sequences to per-protein embeddings which consists of 1024 values (https://colab.research.google.com/drive/1TUj-ayG3WO52n5N50S7KH9vtt6zRkdmj?usp=sharing#scrollTo=tRe7CfuqFFmY, accessed June 2023). To prevent overfitting, we applied a Principle Component Analysis (PCA) and retained the first 20 principal components from the 1024 embedding dimensions. (See “Prediction method” section) These 20 principal components based on embeddings (Embs20) are considered a separate approach to input features to the *aac* features for subsequent training.

#### Generation of pseudo amino acid-based input features

To obtain an alternative representation of protein sequences, bridging the simplicity of amino acid composition with the ability to encode local sequence-order effects, we extracted features based on Amphiphilic Pseudo Amino Acid Composition (APAAC) [60]. APAAC was selected due to its capacity to incorporate hydrophobicity and hydrophilicity correlation factors along the protein chain, potentially capturing biophysical signals relevant for toxin prediction.

We used the iFeatureOmega software suite (https://github.com/Superzchen/iFeatureOmega-CLI/blob/main/README.md, accessed June 2025) [61] to compute APAAC descriptors. The APAAC calculation was configured with the default weight parameter $w = 0.05$, which balances the contribution of basic amino acid composition and the physicochemical sequence-order correlation. To increase the expressiveness of the representation, the correlation tier parameter $\lambda$ was increased from the default (3) to 10, resulting in a 40-dimensional feature vector per protein.

To reduce potential overfitting and ensure consistency in feature dimensionality across input types, we applied Principal Component Analysis (PCA) to the APAAC features and retained the top 20 principal components (APAAC20) for subsequent classifier training.

### Prediction method

We applied several supervised machine learning algorithms to predict toxins. The use of embeddings outsources computationally expensive pre-training, allowing us the use of computationally less demanding algorithms such K-Nearest Neighbors (kNN), Logistic Regression (LR), Support Vector Classifier (SVC). Random Forest (RF) and extreme Gradient Boosting (XGB). We scaled the input features for kNN, LR, and SVC using a Standard Scaler. No scaling was performed for RF and XGB, as these models are not affected by the feature scale. Embeddings contain 1024 numerical dimensions, and our dataset contains after redundancy reduction, approx. 1000 samples for each class. To avoid overfitting, we then reduced the dimensionality of the embeddings by PCA to the first 20 Principal Components, based on learning curve performance across multiple dimensionalities (not shown), and retained 56.46% of the total variance in the embedding space. To prevent data leakage between the training, validation and test set, we used a pipeline to carry out all steps. The pipeline fits all mentioned pre-processing steps to each fold of the training set and then performs the transformations on validation and test data. Model hyper-parameters were optimized via Gridsearch on the training set. Tables 2-6 in the SOM summarize which hyper-parameters were searched and the respective search spaces. The search space was set up on a logarithmic scale around the default values as described in the sklearn documentation (1.5.1). Any hyper-parameters not specifically mentioned were set to default. The hyper-parameter performance was measured by 10-fold cross-validation using Matthews Correlation Coefficient (MCC), a balanced performance metric suitable for binary classification tasks with mild to moderate class imbalance [62, 63]. The architecture’s hyperparameter combination with the highest cross-validation score was selected. The performance on an unseen dataset was measured using a holdout test set, which was kept out of feature transformation and parameter optimization. No further model changes were made on the basis of the test set performance. For details on program and package versions see file *environment.yml* in the data repository.

### BLAST

As a second baseline, we found the closest match in sequence similarity using Blastp in the command line tool from NCBI [64]. We first constructed a custom BLAST database out of the training sequences and then ran the established test set sequences as query, choosing the query with the lowest e-value. The labels from resulting matches were compared to the ground truth labels of the test sets.

### Foldseek

As an additional structural baseline, we used Foldseek [65] to identify the closest structural match for each test protein. First, protein structures were predicted using ColabFold with almost all default settings. The –stop-at-score parameter was set to 85 to reduce runtime while maintaining high-confidence predictions, based on expert recommendation. MSAs were generated using MMseqs2 with the February 2023 release of UniRef30 and the ColabFold environmental database. Once the structure predictions were completed, we used Foldseek [65] to construct a structural database from the training set PDB files. Each test set structure was then queried against this database, and the closest match was identified using Foldseeks default parameters. The predicted label for each test set sequence was taken from its best structural hit, based on the Foldseeks reported metric of probability of homology *prob*. These labels were then compared to the ground truth annotations to assess classification performance.

### Performance measures

We evaluated the model performance on a variety of metrics. We followed the common practice of labeling true positives as (TP), false positives (FP), true negatives (TN) and false negatives as (FN). TP are proteins that are correctly predicted as toxic. FP are the proteins that are wrongly predicted as toxins. TN are proteins that are correctly predicted as non-toxic, and FN are toxins not classified as such.

#### Performance metrics during model optimization

As previously mentioned, we used the Matthew’s Correlation Coefficient (MCC) to measure model performance during hyper-parameter optimization and model selection. MCC was picked for its balanced penalty for FP and FN classified observations and its robustness in imbalanced classification settings [62, 63]. MCC remains informative when class distributions are uneven and both types of misclassification carry different practical costs. MCC was calculated using Eq. 2.

2 $$\begin{aligned} MCC= \frac{TP*TN-FP*FN}{\sqrt{(TP+FP)* (TP+FN)*(TN+FP)*(TN+FN)}} \end{aligned}$$

where:

$$\begin{aligned} TP & = \text {True Positive}\\ TN & = \text {True Negative}\\ FP & = \text {False Positive}\\ FN & = \text {False Negative} \end{aligned}$$

#### Performance metric of final predictor

For better comparability with other predictors additional performance metrics to the MCC were calculated on the finished predictors using the hold-out test set. They include accuracy (Eq. 3), precision (Eq. 4), recall (Eq. 5), ROC-AUC. Bootstrapping was applied to receive a more reliable metric that takes outliers in the data into account. Each metric was averaged across 10000 samples that were chosen at random with replacement. The equations for each metric are listed below with N being the number of bootstrapping samples, and b for the index of each bootstrap-sample.

3 $$\begin{aligned} \overline{Accuracy}= \frac{1}{N}\sum \limits _{b=1}^{N}\frac{TP_b+TN_b}{TP_b+FP_b+TN_b+FN_b} \end{aligned}$$

4 $$\begin{aligned} \overline{Precision}= \frac{1}{N}\sum \limits _{b=1}^{N} \frac{TN_b}{TN_b+FP_b} \end{aligned}$$

5 $$\begin{aligned} \overline{Recall}= \frac{1}{N}\sum \limits _{b=1}^{N} \frac{TP_b}{TP_b+FN_b} \end{aligned}$$

To get the 95% confidence interval for each metric, the Standard Error $SE_{metric}$ was multiplied by 1.96.

6 $$\begin{aligned} SE_{metric}= \sqrt{\frac{\sum \nolimits _{b=1}^{N}(metric_b-\overline{metric})^2}{N}} \end{aligned}$$

7 $$\begin{aligned} CI_{metric}= \overline{metric}+-SE_{metric} *1.96 \end{aligned}$$

With $CI_{metric}$ as the confidence interval of a metric, N being the number of bootstrapping samples, $metric_b$ being the metric of choice for a single bootstrap sample b and $\overline{metric}$ as the mean across all bootstrap samples.

### Investigating the robustness of the models

#### Evaluation of signal peptide bias

Both raw datasets of toxins and non-toxin consist of secreted proteins. We therefore investigated the impact of signal peptides on the performance of our predictor. For this, the SignalP-6.0 [66] was used on both datasets, setting the model mode to “fast”, and the organism option to prokaryotes (“other”). We removed the predicted signal peptides from the original amino acid sequences in our test set. We then applied the same methodology outlined in Generation of embeddings based input features and Generation of vectors of amino acid composition sections to the truncated test sequences.

#### Evaluation of length bias

To investigate the impact of sequence length, we scrambled all sequences and obtained new embeddings using ProtT5. For the scrambling, an out-of-bag sampling with no replacement was done creating a new artificial sequence. For each sequence we generated embeddings, and used them to retrain the best performing model using Support Vector Classifier and the first 20 Principal Components of a PCA. Through shuffling, the new embeddings are restricted to information of sequence length and amino acid composition. To evaluate the effect of length information on training with embeddings, we compared this re-trained model to our baseline (*aac*)- which only uses amino acid composition as input and contains no length information.

### Predictor scope - generation of additional datasets

Some bacterial toxins are known to originate from bacteriophages. We therefore tested Exo-Tox on a dataset of bacteriophage proteins. Our bacteriophage set combines four previously published phage datasets. This includes: the PhaNNs database [67], phage accession IDs from the EMBL Database (European Nucleotide Archive (ENA) (RRID:SCR_006515), European Bioinformatics Institute (RRID:SCR_004727), the full Actinobacteriophage Dataset [68] and a dataset from Zhang et al. [69]. We removed all proteins labeled as fragment, partial sequences or prophage. Prophages were removed, because their sequence is affected by the evolution of the organism’s DNA in which they are embedded, which can change the uniqueness as toxin. We further reduced the set to 30 percent sequence similarity.

To investigate the limits of the specialized predictor Exo-Tox, which was trained on secreted proteins only, we tested the model on other bacterial proteins. For this we chose all proteins from the reference proteomes as published under NCBI: https://www.ncbi.nlm.nih.gov/datasets/genome/ (Accessed in Jan, 2024) from the same species IDs that were found for the bacterial toxins. Any sequences that were part of our exotoxin set were removed. Again, we removed any fragments or partial sequence. In addition any sequences that were already identical and reduced the redundancy to 30% sequence similarity.

To investigate the possibility of unidentified toxins in the control set, we performed an extensive search using regular expressions applied to the protein descriptions provided by the fasta files. Regular expressions were used to identify proteins with descriptions indicative of toxin-like activity. The list of regex terms used in this analysis is detailed in the Appendix C (see Tables 10 and 11).

### Use of generative AI

Each section was first researched, drafted and written by the authors. Sections of the manuscript were then passed to language models including DeepL Write, ChatGPT version 4o, and Anthropic version Heiku to correct grammar, revise code, restructure arguments for better clarity, and checking spelling mistakes.

## Results

### Embeddings distinguish exotoxins from non-toxins

**Fig. 1:**
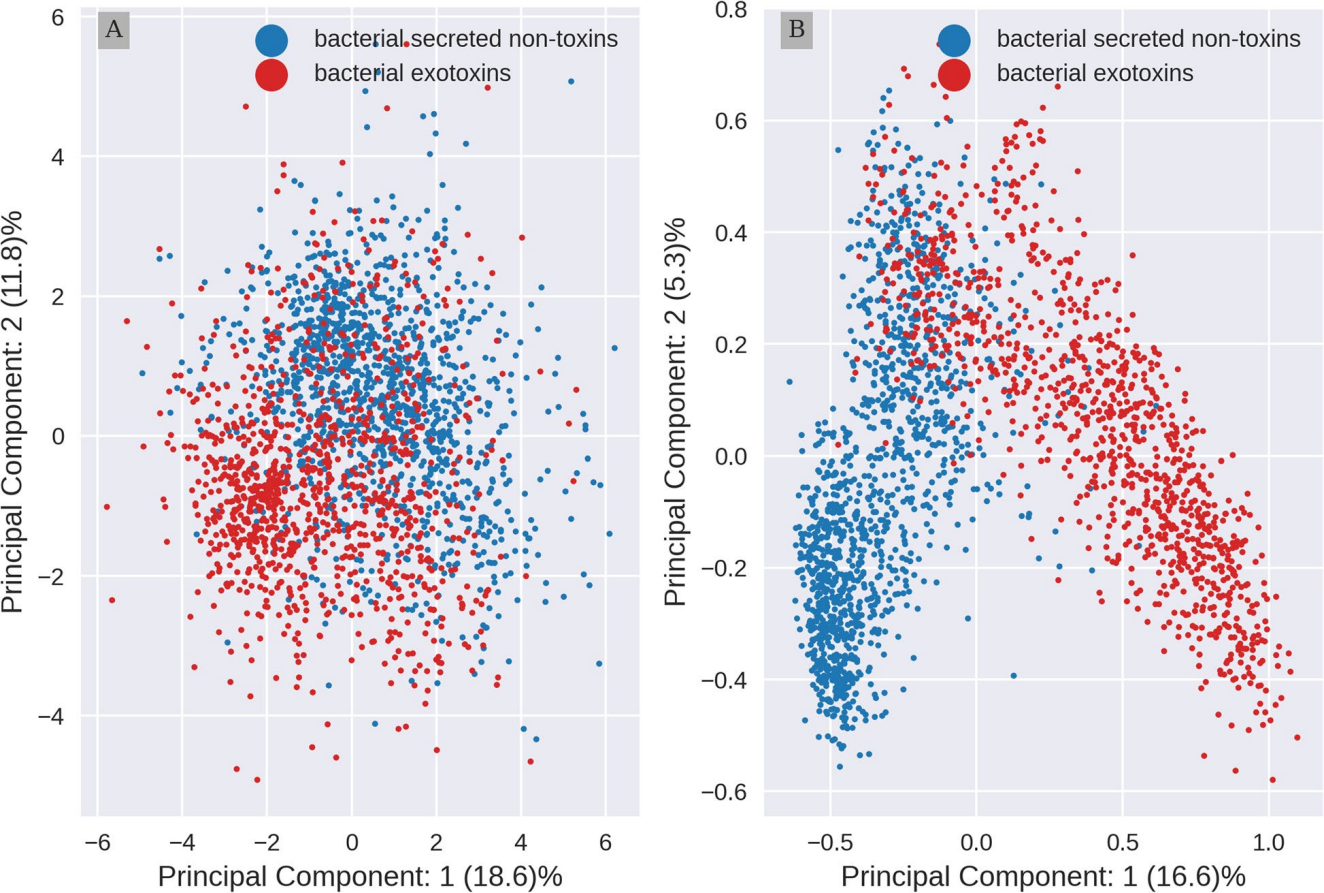
Embeddings information separates better toxins from non-toxins than aac. **A** 2D Principal Component Analysis(PCA) projection of amino acid composition (aac) (**B**) and per protein embeddings by ProtT5. The aac was scaled before the PCA was carried out, embeddings were not scaled (see Material and methods). Bacterial exotoxins (red) and secreted bacterial non-toxins (blue) form overlapping clusters

The relative percentage of each amino acid and their order, defines the physico-chemical properties and functions of proteins. In previous work, we have shown that secreted bacterial proteins and exotoxins have different amino acid usage [56]. However, amino acid composition (aac) cannot capture the global context between amino acids. Protein embeddings created by Protein Language Models (pLM) such as ProtT5, are designed to capture this global context. To explore if aac and protein embeddings distinguish between bacterial exotoxins and bacterial secreted non-toxins, we applied a Principle Component Analysis (PCA) to the data. The first two principal components are visualized in 2D plots (Fig. 1).

This visual analysis shows both representations of proteins form clusters, detecting differences between toxins and secreted non-toxic proteins (control). For *aac*, PCA (Fig. 1A) uncovers two disperse clusters with extensive overlap along the whole range of the second principal component (PC 2). In contrast, for embeddings (Fig. 1B) the clusters are more compact, with a small overlap limited to the upper half of the PC 2. These results indicate that representation of secreted proteins and toxins in embeddings capture more information than *aac*, possibly allowing for a better separation of the two classes of proteins when creating a predictor model.

### Embeddings based prediction outperforms *aac*

Both representations detect differences between secreted and toxic proteins. Therefore, we evaluated if the information contained in them is enough to predict toxicity potential of secreted proteins by training a series of simple supervised models. We compared three approaches of input features. A naive approach using the aac as input, a second method using dimensionality reduced embeddings (Embs20). We also added a third approach using dimensionality reduced Amphiphilic Pseudo Amino Acid Composition (APAAC20) combining amino acid composition with sequence based features. These machine learning (ML) approaches were compared to two baselines: BLAST and Foldseek. BLAST alignment as classification method does not rely on machine learning, but rather finds motifs of sequence similarity between proteins. Foldseek is a more recent alignment tool that leverages protein structures.

**Fig. 2:**
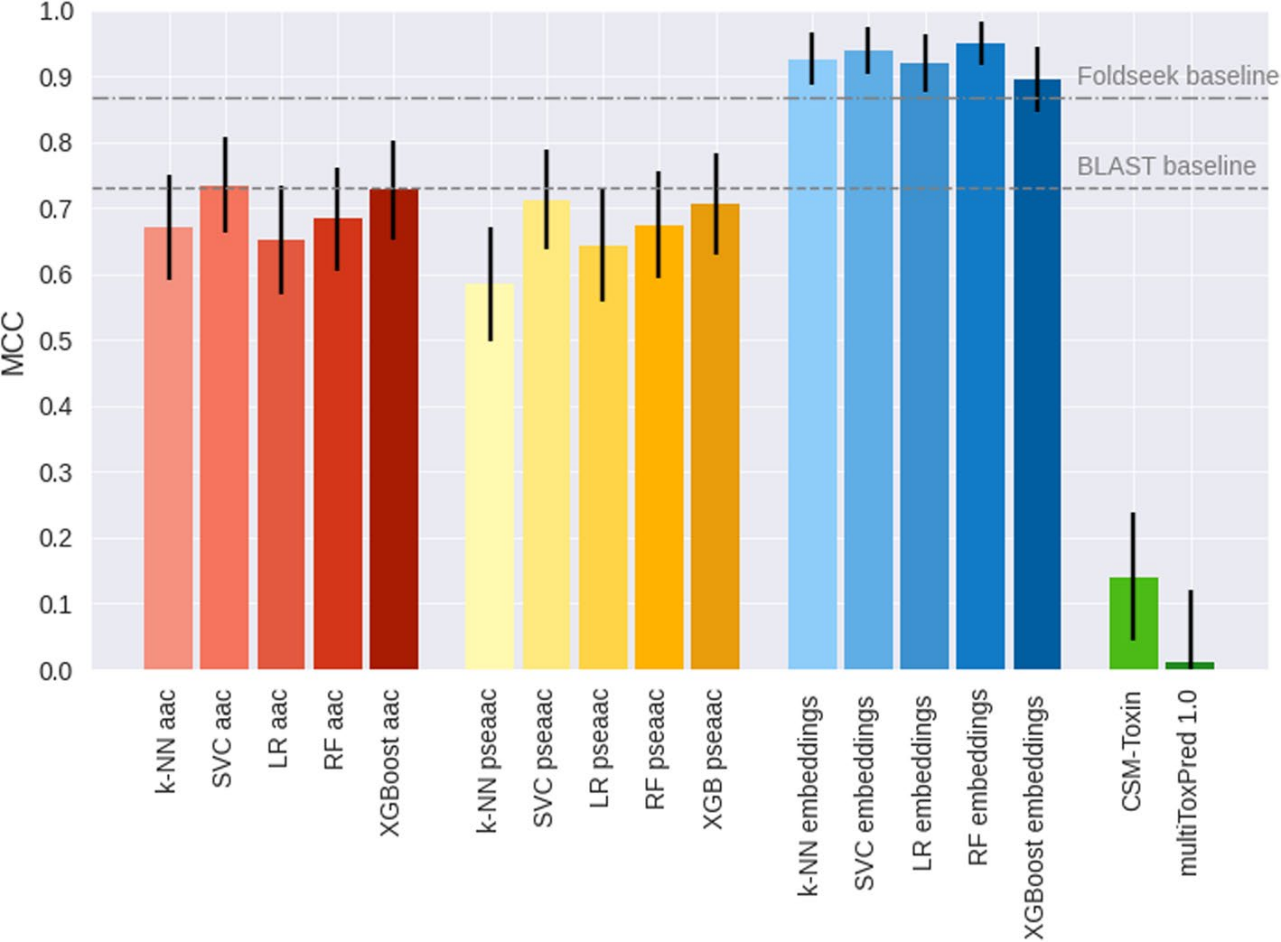
Toxicity of secreted proteins predicted accurately from embeddings. Different exotoxins prediction methods are compared. This includes three sets of input features. Methods that use the amino acid composition as input are depicted in shades of red. Methods using the first 20 Principle Components that retain the most information from Pseudo Amino Acid Composition are shown in hues of yellow. Methods using the first 20 Principle Components that retain the most information from ProtT5 protein embeddings are in shades of blue. The different input features approaches are compared to BLAST and Foldseek as a baselines (gray lines) and CSM-Toxin [47] and MultiToxPred 1.0 [48], two state of the art generalized toxin predictor not specialized on bacterial proteins (green). Data: hold-out test set of bacterial exotoxins and bacterial, secreted non-toxins with less than 30% sequences similarity to training set of CSM-Toxin and training set of our proposed predictor. Metric: Matthew’s correlation coefficient (MCC). Model architectures: kNN: K-Nearest Neighbors, LR: Logistic Regression, SVC: Support Vector Classifier, RF: Random Forest and XGB: Extreme Gradient Boosting. The model architectures are differentiated by color intensity. From light to dark: kNN, LR, SVC, RF, and XGB. Black whiskers mark the 95% interval with ± the 1.96 the standard error

Five machine learning architectures were tested. They included K-Nearest Neighbors (k-NN), Support Vector Classifier (SVC), Logistic Regression (LR), Random Forest (RF) and extreme Gradient Boosting (XGB). In all five, Embs20 outperformed *aac* and the (APAAC20) as input feature (Fig. 2). Differences based on the type of input used, are statistical relevant when evaluating the 95% confidence interval(CI). In contrast, differences in architecture are negligible, as interpreted by the overlapping of CI ranges for both inputs sets for kNN, SVC, LR, RF and XGB.

All metrics for the evaluation of all methods are in Table 1. BLAST performed similar to the *aac* and APAAC20 approaches, with MCC of 0.73 and accuracy of 0.86. For precision and recall, however, the performance ranking changed. BLAST recall of 0.71 is below the naive *aac* method with values between 0.78 and 0.87. This suggest that BLAST has a higher probability to miss true toxins.

**Table 1:** Embeddings-based predictor outperformed other approaches

Method	MCC	Accuracy	Precision	Recall	ROC AUC
BLAST	0.731± 0.066	0.858± 0.038	0.972± 0.031	0.708± 0.074	-
Foldseek	0.866± 0.053	0.934± 0.027	0.961± 0.033	0.885± 0.052	-
**Amino Acid Composition (*aac*)**					
kNN	0.670± 0.080	0.837± 0.040	0.836± 0.061	0.796± 0.064	0.919± 0.029
SVC	0.735± 0.073	0.868± 0.036	0.843± 0.057	0.871± 0.054	0.950± 0.020
LR	0.652± 0.082	0.828± 0.040	0.828± 0.062	0.783± 0.066	0.906± 0.034
RF	0.683± 0.079	0.843± 0.039	0.830± 0.060	0.824± 0.061	0.931± 0.026
XGB	0.727± 0.075	0.865± 0.037	0.851± 0.058	0.851± 0.058	0.942± 0.023
**Pseudo Amino Acid Composition (APAAC20)**					
kNN	0.585± 0.087	0.794± 0.044	0.824± 0.066	0.695± 0.073	0.879± 0.037
SVC	0.712± 0.076	0.856± 0.038	0.826± 0.059	0.866± 0.055	0.923± 0.030
LR	0.644± 0.085	0.823± 0.043	0.793± 0.065	0.825± 0.060	0.889± 0.036
RF	0.674± 0.081	0.838± 0.040	0.815± 0.062	0.832± 0.060	0.928± 0.028
XGB	0.712± 0.076	0.856± 0.038	0.827± 0.059	0.865± 0.056	0.922± 0.029
**Embeddings (Embs20)**					
kNN	0.926± 0.040	0.963± 0.020	0.941± 0.037	0.980± 0.023	0.986± 0.012
SVC	0.938± 0.036	0.969± 0.019	0.948± 0.035	0.986± 0.019	0.995± 0.006
LR	0.919± 0.043	0.960± 0.021	0.953± 0.034	0.959± 0.032	0.994± 0.005
RF	0.950± 0.034	0.975± 0.017	0.973± 0.026	0.973± 0.026	0.992± 0.007
XGB	0.895± 0.049	0.948± 0.024	0.933± 0.040	0.953± 0.034	0.981± 0.014
CSM-Toxin	0.140± 0.097	0.571± 0.054	0.785± 0.223	0.075± 0.042	-
MultiToxPred 1.0	−0.066±0.111	0.490± 0.056	0.402± 0.102	0.247± 0.073	-

Matthew Correlation Coefficient (MCC), Accuracy, Precision, Recall, and Receiver Operating Characteristic - Area Under the Curve (ROC-AUC) for different exotoxin prediction approaches. Methods include BLAST, Foldseek, the CSM-Toxins [47] and MulitToxPred 1.0 [48] predictors, and three different input feature approaches paired with five machine learning architectures. Input features are the amino acid composition (*aac*),the first 20 Principal Components of a Principal Component Analysis of pseudo amino acid composition (APAAC20) and the first 20 Principal Components of a Principal Component Analysis performed on T5 per-protein embeddings (Embs20). Architectures include kNN (K-Nearest Neighbors), LR (Logistic Regression), SVC (Support Vector Classifier), RF (Random Forest), and XGB (Extreme Gradient Boosting). The models paired with Embs20 input features show the best performance across all metrics

Foldseek outperformed both the *aac* and *APAAC20* feature-based models across all evaluation metrics, achieving an MCC of 0.87. While Foldseek performed strongly, the embedding-based approach (Embs20) showed a consistent trend toward higher performance across metrics, including MCC, accuracy, precision, and especially recall (see Fig. 2). Embs20 and Foldseek share comparable performance when comparing the 95% confidence intervals for MCC, accuracy, and precision. Notably, recall remains statistically higher in the embedding models even under the more stringent double standard error interval, underscoring their superior ability to detect true positive toxins.

As benchmarks for the predictor, we used two available predictors: CSM-Toxin [47] and MultiToxPred 1.0 [48]. CSM-Toxin is a state of the art general toxin predictor, trained on a set including animal and bacterial toxins. Using our test set in the CSM-Toxin predictor gave a surprisingly low MCC score (0.14). The low value is reflected by the CSM predictions, where it correctly identified 174 out of 177 non-toxins, but missed the majority of the bacterial toxins by identifying 11 out of 147.

The second benchmark, MultiToxPred 1.0 [48], is a model designed to classify sequences into multiple toxin types or as non-toxins, without a restriction to a particular kingdom or source. MultiToxPred 1.0 yielded a MCC of −0.066±0.111 on our test set. This value is statistically indistinguishable from zero, indicating that the classifier performed no better than random guessing. The model correctly identified 36 exotoxins, but also misclassified 53 out of 177 non-toxins as toxins.

As both the APAAC20 and the *aac* based methods performed similarly, subsequent performance analysis focused on embeddings and *aac* alone.

### Signal peptides do not influence prediction

**Fig. 3:**
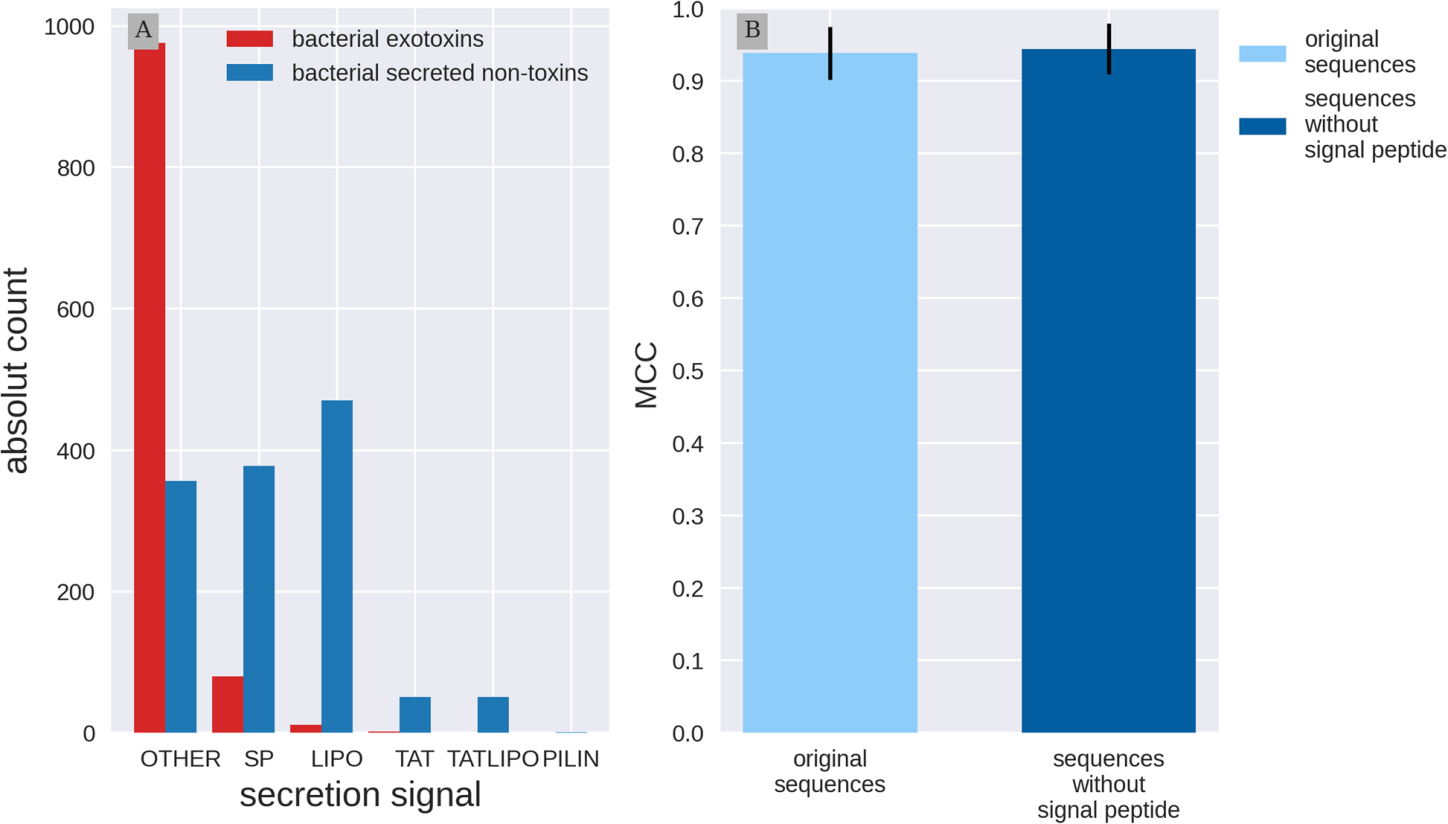
Signal Peptides do not influence toxin prediction Panel A: Exploration of signal peptides predicted with SignalP-6.0. Bacterial exotoxins are in red, the bacterial secreted non-toxins are in blue. Signal Peptides distinguish between two translocation routs Sec and Tat and three Signal Peptidases SPI-III. Prediction include SP: Sec/SPI, LIPO: Sec/SPII, TAT: Tat/SPI, LIPOTAT: Tat/SPII, PILIN: Sec/SPIII and OTHER indicates no known signal peptides. The majority of exotoxins does not have a predicted signal peptide. Panel B: Performance comparison with and without signal Peptides. Model architecture was introduced in Fig. 2 Support Vector Classifier (SVC) using the first 20 Principal Components calculated on per protein protT5 embeddings (Embs20). Two versions of the test set are compared. Light blue are the original test set sequences. Dark blue: the test sequences without the predicted signal peptides. Embs20/SVC performs equally well on both test set versions

All sequences in this study are secreted proteins, but not all toxins have traditional signal sequences. We investigated which secretion signals are present in the data using SignalP-6.0. Signal Peptides distinguish between two translocation routs: Sec and Tat and three Signal Peptidases: SPI-III. SignalP includes SP: Sec/SPI, LIPO: Sec/SPII, TAT: Tat/SPI, LIPOTAT: Tat/SPII, PILIN: Sec/SPIII and OTHER indicates no known signal peptides. We found that the presence of signal peptides differs between the toxins and non-toxins. For over 90% of the toxins no known secretion signal was identified by SignalP-6.0, while only 27% of the non-toxins contained no predicted secretion signal (see Fig. 3, Panel A). The most frequent signal peptides were the sec signals SP (378) and LIPO (470) occurrences in the control proteins. Because of this imbalance, and the fact that context is captured by the embeddings, we investigated if signal sequences could be introducing a bias in the Embs20 predictor.

Therefore, we removed the predicted signal peptides from the test set sequences and obtained new embeddings to evaluate the performance once again. We compared the predictor performance on the original and modified test sets using the Embs20/SVC model architecture (Fig. 3B). Embs20/SVC showed similar results on both sets, with no significant differences in the measured metrics, as the MCC shows (for both sets the MCC is around 0.94). This suggests that the signal peptide has little influence on the information captured by the embeddings and, therefore, has no effect in the predictors ability to identify toxicity in secreted bacterial proteins. Additional metrics are listed in Table 4 in Appendix A.

### Protein length does not influence prediction

Previously, we identified differing median sequence lengths between our two sets [56], raising concerns about potential bias. To examine the impact of these length variations, we retrained our best performing model (Embs20/SVC) on scrambled sequences. Their embeddings should reflect protein length and amino acid composition, but a different context. We compared this retrained model to our baseline which solely captures the amino acid composition, but not the protein length. (see baseline in Fig. 2). Both methods without context information yield comparable performance across all metrics, including an MCC of around 0.7 (see Table 2). The consistent results across accuracy, precision, recall, and ROC-AUC suggest that the protein length doesn’t skew exotoxins predictions in the Embs20/SVC model, allowing us to assume that the information included in the embeddings used by the Embs20 model is not influenced by protein length.

**Table 2:** Protein length does not influence prediction

	MCC	Accuracy	Precision	Recall	ROC AUC
aac/SVC original sequences	0.735± 0.073	0.868± 0.036	0.843± 0.057	0.871± 0.054	0.950± 0.020
Embs20/SVC scrambled sequences	0.685± 0.078	0.840± 0.040	0.788± 0.062	0.885± 0.052	0.908± 0.031

Matthew Correlation Coefficient (MCC), Accuracy, Precision, Recall, and Receiver Operator Curve - Area Under the Curve (ROC-AUC) for two different inputs. Methods include the Embs20/SVC trained on randomly scrambled sequences and the aac/SVC predictor that was introduced in Table 1

### Predictor limitations

We know that the embedding-trained predictor (Emb20/SVC) has been trained with context information, and that length and signal peptides information have not introduced a bias in the embedding-based selection process. The next step is to investigate the limitations of this model and it applicability to a new group of proteins. For this, we tested Embs20/SVC on bacteriophages, and on a set of bacterial proteins independent of their secretion status. (For details on the preprocessing of both sets, see Materials and methods). Bacteriophages were chosen as many lysogenic phages contain toxin genes (prophages). The predictor classifies 159637 bacteriophage proteins as toxins (97%) and 54903 (63%) of bacterial proteins as toxins. (See Table 3). For control of performance, we also tested our full dataset of training and testing combined. In this case, 2360 (98%) of the toxin data set is correctly classified as toxins, while from the secreted bacteria proteins only 67 (0.007%) were wrongly classified as toxins.

**Table 3:** Datasets and predictor results

Dataset	Toxin (predicted)	Non-toxin (predicted)
**Embs20/SVC**		
phages (reduced)	159637	3787
bacterial control (reduced)	54903	31273
original exotoxins	2360	36
secreted non toxins	67	9015
**aac/SVC**		
phages (reduced)	127344	36080
bacterial control (reduced)	54316	31860
original exotoxins	2150	246
secreted non toxins	700	8382

Predictions obtained for exotoxins and secreted non-toxins using Embs20/SVC (embedding-based) and aac/SVC (amino acid composition-based) classifiers across different datasets.

For comparison, the same analysis was performed using our best naive model based on amino acid composition (*aac*/SVC). In this case, 127344 (78%) of phage proteins are classified as toxins, and from the set of general bacterial proteins, 54316 (63%) were classified as toxins, which are similar to the results yielded from Embs20/SVC. The classification of exotoxins and secreted bacterial proteins set gave the expected results with 2150 (90%) of the toxins, and only 700 (8%) of secreted proteins identified as toxins. Although the toxin and secreted proteins sets are mostly correctly classified, results with bacteriophage proteins and bacterial proteins are unexpected.

### Common classification by both predictors

**Fig. 4:**
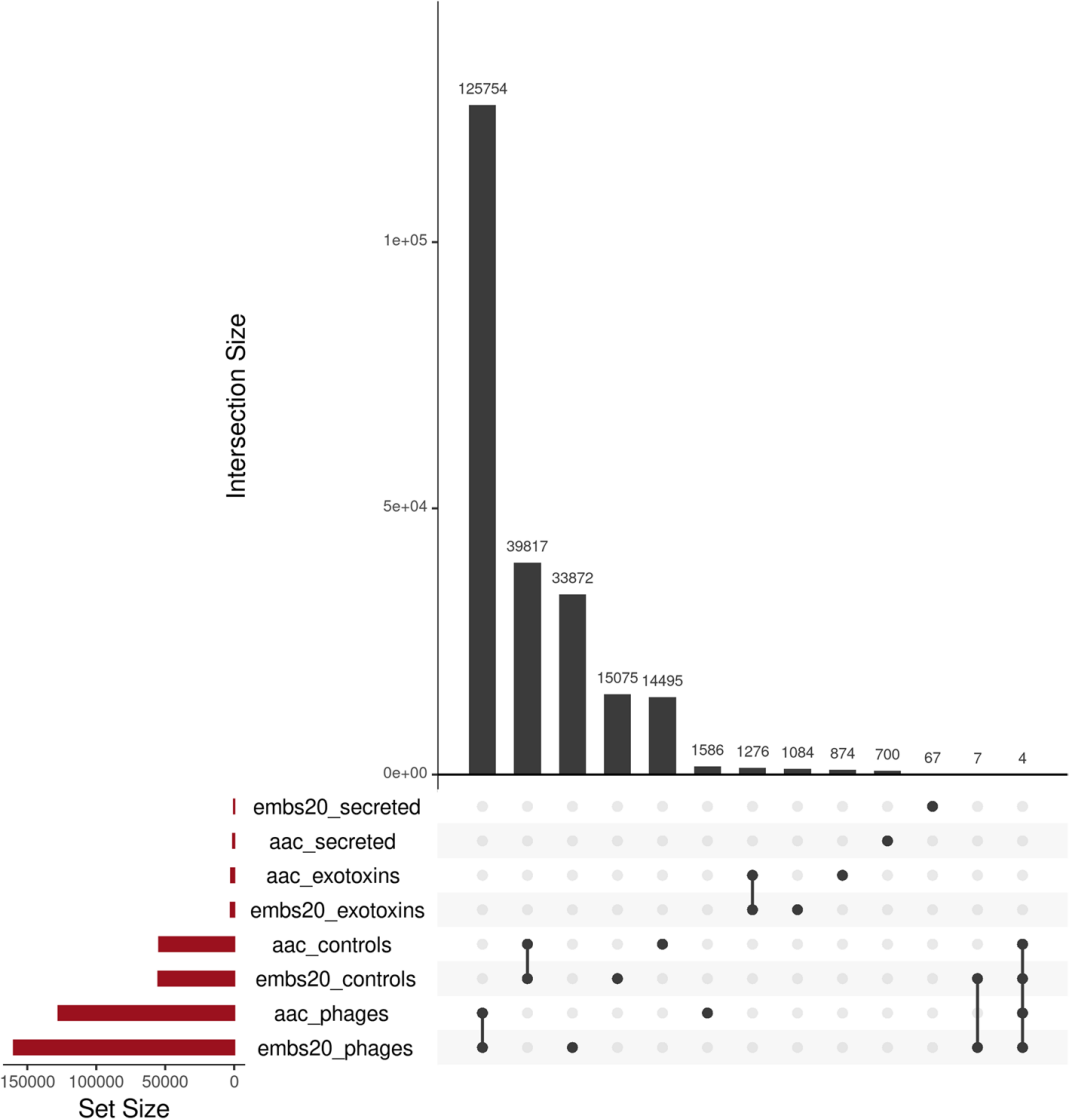
Several proteins are identified as toxins by both predictors, but not all. Size of the sets is shown in the lower left corner. The intersection of each set’s prediction are shown by the dots and bars located below each of the bars on the bar plot above. Four sets were tested 1) secreted: secreted bacterial proteins, 2) exotoxins: bacterial exotoxins, 3) controls: bacterial proteins independent of secretion status and 4) phages: phage proteins not containing prophages. Two predictors were tested 1) aac: was trained on amino acid composition and 2) Embs20: was trained on the first 20 Principle Components of per-protein embeddings

Because of the previous results of the predictor on bacteriophage and general bacterial set, we examined which proteins were classified as toxins by both, the amino acid based (*aac*) and the embeddings-based (Embs20) predictor. The Upset plot allows us to visualize the different results and the number of common proteins classified by the predictors (Fig. 4). The *Set Size* reflects the differences or similarities in number of proteins classified as toxins. The *Intersection Size* allows to see the numerical value of proteins in the evaluated set or intersection of sets, the dots indicate which set is being evaluated, and the lines connecting different sets show between which sets the proteins intersect. From all datasets, 125764 phage proteins are classified as toxins by both models (78% of Embs20/SVC, 98,7% of aac/SVC), and 39817 control bacterial proteins (“controls”) (78% of the Embs20/SVC toxins, 73% of the aac/SVC toxins). While 1276 exotoxins were classified as toxins by both models (54% of the Embs20/SVC, 59% of the aac/SVC), none of the proteins from the secreted dataset were identified as toxins by both models.

## Discussion

Many toxin classifiers have previously been created. Their development has morphed from highly specialized to very broad. Specialized predictors focused on specific biological kingdoms [25–27], defined protein length [25, 28, 30–33]. Others focus on effector proteins [37, 38], virulence factors [40, 42–45] or were associated with distinct physiological activities [70]. Newer models are often trained on multi-species data sets [47–49, 71, 72] or exclusively on animal toxins [73]. While their wide focus may not always be explicitly stated in the literature, it can be inferred from the absence of constraints tied to specific use-cases. Some of these broader models have shown poor performance for domain-specific challenges, such as bacterial exotoxins [74].

To close this gap, we created predictors specialized in the classification of secreted bacterial exotoxins. The best performing model, Embs20/SVC, was renamed Exo-Tox. Unlike other predictors, Exo-Tox avoids the inclusion of virulence factors and endotoxins. It also narrows its training scope to secreted bacterial proteins to enhance performance within this specific domain. By selecting proteins from the same kingdom and sub-cellular localization, we aimed at minimizing extraneous features that separate the toxins and controls.

Considering the recent application of Natural Language methods for the creation of protein Language Models, Exo-Tox uses ML algorithms used for protein representation by embeddings using global context information that can be found in the sequence of proteins [54]. With Matthews Correlation Coefficient (MCC) of above 0.9, Exo-Tox provides a reliable tool in distinguishing secreted bacterial exotoxins from secreted non-toxins.

The embedding-based approach implemented in Exo-Tox outperformed both naive amino acid composition (*aac*) and the Amphiphilic Pseudo Amino Acid Composition (APAAC20) method (Table 1). This suggests that protein-level toxicity signals are better captured by global contextual representations learned from large-scale language models (per protein embeddings) than by simple compositional or physicochemical summaries. In particular, embeddings encode long-range interprotein residue dependencies (unseen by *aac*) and global sequence features that cannot be represented by local pattern (unseen by Blast).

A pseudo amino acid composition was included following a reviewer’s suggestion, with the hypothesis that it could bridge the gap between *aac* and deep learning embeddings, as it incorporates both amino acid composition and global sequence-order features based on physicochemical properties. Surprisingly, APAAC20 did not outperform simple *aac* in our benchmarks. The lack of improvement may be due to the fact that the *aac* representation already captures critical discriminatory information in our dataset. In our previous work [56], we demonstrated that specific residues (such as cysteine and histidine) are significantly overrepresented in exotoxins, suggesting that compositional signals alone may be particularly informative in this domain. Thus, while APAAC20 adds theoretical complexity, it does not necessarily increase predictive power in this specific toxin classification task.

Exo-Tox also outperformed the BLAST baseline in nearly all performance metrics, including MCC, accuracy, and recall. Only in precision did BLAST match the embedding-based predictor. This reflects a core strength of Exo-Tox: its ability to generalize beyond sequence-local homology and identify toxicity signals in proteins that diverge significantly in primary sequence. In practice, the choice of tool depends on the application: for discovery pipelines where validation cost is high, precision becomes the priority, and both BLAST and Exo-Tox are suitable. However, in medical or biosecurity contexts where missing a toxin is unacceptable, Exo-Tox’s high recall makes it a better choice.

When comparing Exo-Tox to the Foldseek baseline, we observe a similar overall performance: Foldseek achieved an MCC of 0.866 ± 0.05, while Exo-Tox scored 0.950 ± 0.034. Although the 95% confidence intervals of both methods slightly overlap, implying that the difference is not statistically significant, the magnitude of the difference and the minimal overlap suggest a trend favoring Foldseek in overall classification accuracy. However, in toxin recall, Exo-Tox outperforms Foldseek, making fewer false negatives and better capturing the positive class.

The similar performance between these fundamentally different approaches is notable. Foldseek encodes protein structure as a discrete sequence over a 3Di alphabet derived from 3D backbone interactions and performs alignments based on structural geometry [65]. Embeddings, on the other hand, are derived from the amino acid sequence alone, yet have been shown to also encode structural information [52, 75, 76]. This convergence suggests that both representations may capture overlapping aspects of protein function. However, because our training and test sets were filtered based only on sequence similarity (at 30% identity), it is possible that shared fold-level features remain. A more rigorous evaluation involving fold-similarity reduction could help determine whether the models truly generalize to novel folds or rely on latent structural redundancy. Such an analysis would clarify to what extent structural similarity drives the observed alignment between Foldseek and embedding-based predictions.

CSM-Toxin [47] and Exo-Tox both use context information from protein sequences encoded by pre-trained protein language models. The challenges of generalizing such predictors became evident when we applied CSM-Toxin to our test set, which includes only prokaryotic toxins. CSM-Toxin achieved an MCC of just 0.14. This performance drop may be related to the composition of its training set, which consists of approximately 90% eukaryotic and only 10% prokaryotic proteins. With limited exposure to bacterial toxins during training, the model appears to struggle to generalize in this domain. In our test set, it correctly predicted most non-toxins (174/177), but only identified 11 out of 147 exotoxins, leading to extremely low recall and a low MCC. These results highlight the difficulty of cross-kingdom prediction when the training data is heavily skewed toward eukaryotic toxins. Our interpretation is supported by Pan et al. [74], who also observed a significant performance drop (from MCC 0.793 to 0.022) when animal-toxin predictors were evaluated on bacterial toxins. Their conclusion aligns with our own prior findings [56], which showed distinct differences between animal and bacterial toxins in terms of sequence length, isoelectric point, and overall sequence similarity. Together, these results emphasize the importance of domain-specific training data for reliable toxin prediction.

MultiToxPred 1.0 [48] also showed low performance on our bacterial exotoxin test set. This model uses pseudo amino acid composition and dipeptide features to classify toxins across diverse biological origins. While such an approach may be suitable for broad toxin detection tasks, its lower performance in our benchmark likely reflects limitations in both representation granularity and training set composition. In particular, the inclusion of synthetically generated sequences in the negative class, which differ biologically from naturally evolved proteins [77–79], may reduce the model’s ability to generalize to real bacterial proteins. The proportion of bacterial sequences in the training data is not clearly reported, making it difficult to assess how well the model is calibrated for bacterial toxin recognition. In contrast, Exo-Tox was trained exclusively on biologically derived, secreted bacterial proteins with careful curation of both toxin and non-toxin classes. This highlights a broader point: for specialized biological applications, particularly in prokaryotic systems, domain-specific training data are critical. Our findings support previous work showing that general-purpose models can struggle when applied to niche functional categories unless appropriately benchmarked and validated.

We investigated potential biases by signal peptides (SPs) or protein length, and found no significant difference between Exo-Tox and comparative architecture predictors failing to contain this two aspects. SPs are crucial for certain secretion pathways; however, using SignalP, we observed that most toxins lacked identifiable SPs, suggesting alternative secretion systems (like T1SS, T3SS, T4SS, or T6SS). When we assessed bias from SP presence, we truncated SPs from primary sequences and retrained the predictor. Both predictors (with and without SP information) performed equally, confirming that SPs do not bias the training. Additionally, despite prior analysis identifying significant differences in median sequence lengths between groups [56], predictors trained with and without length information performed comparably. This demonstrates that neither SPs nor protein length significantly impact model performance, validating our selection of secreted bacterial proteins as an appropriate negative set.

We tested the transferability of knowledge of Exo-Tox on bacteriophages and bacterial proteins. They were chosen given the relation of bacterial exotoxins with bacteriophages and their usage by bacteria. Bacteriophages are considered the origin of many bacterial exotoxins [80–82]. Exo-Tox classified 97% of bacteriophages proteins as toxins. This is unlikely to be accurate. Current research does not suggest that every phage protein is a toxin. This over-classification can lie in a dataset bias: toxins in our training data disproportionately originate from phages, leading Exo-Tox to associate phage-related patterns with toxicity. In the set of bacterial proteins, including non-secreted ones, Exo-Tox classifed about 60% as toxins. Investigating which proteins were classified both by amino acid composition and which by embeddings, revealed a large overlap. This indicates that amino acid composition alone has a large influence.

A recent publication addresses the poor transferability of the model to biological molecules. They consider that the splitting of the training and test sets is the key for transferability [83]. Although our predictor’s training uses a combination of the randomness and sequence similarity reduction, which are the factors named by the authors, it will be interesting in the future to use their platform for splitting of the sets to determine if this plays a role. As for now we can only hypothesize that i) The embeddings principal components chosen for training are capturing bacteriophage specific information, or ii) the small data set for toxins contain a bias we have not been able to control causing an overfitting of Exo-Tox. Our results show that length and signal sequence are not playing a role in the classification. Whatever the reason, these findings reveal a significant limitation: Exo-Tox is most reliable when used exclusively for distinguishing secreted exotoxins from secreted non-toxins, the domain for which it was trained.

While Exo-Tox demonstrates strong performance in its intended domain, expanding its applicability to all bacterial proteins remains a challenge. One solution will be to expand the training dataset. However, this can be only realized when more experimental backed sequences are available and their labels confirmed. Our efforts to expand our controls are summarized in the Appendix C (see regex Tables 10 and 11), which revealed many potential false negatives. A predictor trained on such data would struggle to achieve high recall for true toxins, as the signal from mislabeled non-toxins could overwhelm the true positive signals. However, removing such entries without them being known toxins, might remove the most interesting edge cases, introducing a bias entirely dependent on the initial data curation. For a truly general predictor that can effectively extrapolate toxicity regardless of origin, training data would need to encompass all biological kingdoms. This would need to include plants, fungi, [84, 85] even bacteriophages. Yet, the practicality of this approach is hindered by the overwhelming abundance of unconfirmed animal and bacterial toxins in existing protein databases. In light of these considerations, we advocate for the development of more specialized predictors, by narrowing the focus to specific applications or biological domains, which should reflect the training data.

In general, Exo-Tox is a specialized predictor for secreted bacterial exotoxins, addressing a key gap left by generalized predictors with cross-kingdom training data. Exo-Tox demonstrates strong predictive performance, outperforming amino acid composition models, BLAST-based approaches, and generalized predictors like CSM-Toxin and MultiToxPred 1.0. We show that potential confounding factors, such as protein length and signal peptides, do not bias the models predictions. While the over-classification of bacteriophage proteins and bacterial proteins independent of secretion status highlight the impact of dataset bias, it also underscores the importance of domain-specific training data and the need for clear application scopes. To support reproducibility and replicability, we provide open access to the model, training data, and usage instructions via the Open Data repository of the LMU Munich: https://doi.org/10.5282/ubm/data.576 and https://doi.org/10.5282/ubm/data.665.

## Data Availability

All code used for data analysis is accessible in the LMU University repository 10.5282/ubm/data.576, and under 10.5282/ubm/data.665 (review process). As stated in the data availability statement, raw data and code for data wrangling that support the findings of this study are openly available in LMU repository.

## Appendix A: signal peptides influence on model performance

**Table 4:** Signal peptides do not influence the model performance

	MCC	Accuracy	Precision	Recall	ROC AUC
Original Sequences	0.938± 0.036	0.969± 0.019	0.948± 0.035	0.986± 0.019	0.995± 0.006
No Signal Peptides	0.944±0.035	0.972± 0.018	0.954± 0.033	0.986± 0.019	0.996± 0.005

***No signal peptides*** represent the values obtained with the model trained on embeddings of sequences without the signal sequence region predicted by SignalP6.0

## Appendix B: hyper-parameters model optimization

List of hyper-parameters that were used to optimize the machine learning models during GridSearch.

**Table 5:** kNN hyperparameters set during GridSearch

Hyperparameter	Values
n_neighbors	1, 3, 5, 7, 9
p	1, 2, 3

Hyperparameters screened during the k-nearest neighbors (kNN) GridSearch during model training

**Table 6:** SVC hyperparameters set during GridSearch

Hyperparameter	Values
C (Regularization Strength)	0.1, 0.3, 1, 3, 10, 30
Gamma (Influence)	0.0001, 0.0003, 0.001, 0.003, 0.01

Hyperparameters screened during the Support Vector Classification (SVC) GridSearch during model training

**Table 7:** Hyperparameters screened during the Logistic Regression (LR) GridSearch during model training

Hyperparameter	Values
C (Regularization Strength)	0.0001, 0.001, 0.01, 0.1, 1, 10
Solver	“liblinear”, “sag”, “saga”, “newton-cg”
Penalty	L2

**Table 8:** RF hyperparameters set during GridSearch

Hyperparameter	Values
Max Features	3, 4, 5, 6
Max Leaf Nodes	25, 50, 75
n_Estimators (Number of Trees)	1000

Hyperparameters screened during the Random Forest (RF) GridSearch during model training

**Table 9:** XGB hyperparameters set during GridSearch

Hyperparameter	Values
min_child_weight	3, 4, 5, 6
max_depth (Max Depth of Tree)	25, 50, 75
gamma (Minimum Loss Reduction to Split)	0.001, 0.01, 1, 10
reg_lambda (Regularization Term)	0.001, 0.1, 1, 10
subsample (Percentage of Observations Used Per Tree)	1
learning_rate (Learning Rate)	0.05, 0.1, 0.2
Objective (Loss Function)	“binary:logistic”

Hyperparameters screened during XGBoost GridSearch during model training

## Appendix C: potential toxin

The regular expression (Regex) search applied to the NCBI-derived control set identified over 1,300 proteins with potential toxin activity based on their descriptions. When cross-referenced with UniProt annotations, only 3.34% of these sequences carried the UniProt KW0800 keyword (indicating toxicity). This mismatch between descriptions and annotations highlights inconsistencies in protein labeling within public databases.

**Table 10:** List of Regex terms part 1 that likely indicate toxicity found in a control set of bacterial proteins

Category	Regex Pattern	Rationale
**Secretion System Effectors**	(?i)(T7SS effector.*toxin) T9SS.*sorting domain T9SS.*target domain	Identifies effector proteins secreted via type 7 and type 9 secretion systems, which are commonly linked to toxins.
**Pore-Forming Toxins**	pore-forming	Identifies toxins that disrupt membranes, potentially forming pores in host cells.
**Chemotaxis Inhibitors**	chemotaxis-inhibiting	Identifies proteins that likely inhibit bacterial chemotaxis.
**Secretion System Proteins**	Dot/Icm secretion system protein	Identifies proteins from the Dot/Icm secretion system.
**Effector Toxins of Types III, IV and VI**	(?i)(effector.*(type III|type IV|type VI|T6SS|T4SS|T3SS|type 3|type 4|type 6)) ((type III|type IV|type VI|T6SS|T4SS|T3SS|type 3|type 4|type 6).*effector)	Identifies effector proteins related to Type III, IV, and VI secretion systems, commonly associated with delivering proteins directly into host cells.
**Known Toxin Subunits**	(?i)(toxin ADP-ribosyltransferase subunit ArtA|cytolethal distending toxin subunit B family protein | two-peptide bacteriocin plantaricin EF subunit PlnE |putative AB5 enterotoxin ADP-ribosylating subunit YtxA|alpha-xenorhabdolysin family binary toxin subunit A|alpha-xenorhabdolysin family binary toxin subunit B|Shiga toxin A subunit|cytolethal distending toxin subunit B family|Shiga toxin 2 subunit A)	Identifies known toxic subunits of large, multi-component toxins.
**General Toxin Keywords**	(?i)(toxin/[|toxin,|toxin;|toxin protein|toxin peptide| toxin B (plasmid)|toxin 1|toxin 2| toxin 5|toxin of|toxin component|toxin-like|toxin-type| toxin*.family|toxin*.domain| family*.toxin| pre-toxin|polymorphic toxin)	Captures generic mentions of “toxin” in protein descriptions, including common family or component annotations. The regex search for the keyword “toxin” on its own is not sufficient due to component words like e.g.: “antitoxins”, which are not toxins.
**Colicin and Bacteriocins**	colicin-like pore-forming|colicin-like bacteriocin| bacteriocin/[| bacteriocin,| bacteriocin.*domain| bacteriocin.*family| bacteriocin-like| bacteriocin class| bacteriocin fulvocin C-related	Identifies colicin-like and bacteriocin-related proteins.
**Hemolysins and Leukocidins**	hemolysin/[| hemolysin domain| hemolysin.*family| hemolysin-type| hemolysin*.precursor| hemolysin-type| leukocidin	Identifies probable hemolysins and leukocidins.
**Cytotoxins and Cytolysins**	cytotoxin/[|cytotoxin.*domain| cytotoxin.*family| cytolysin| cytotoxix	Captures probable cytotoxins and cytolysins.
**Pyrocines**	pyocin/[| pyocin.*domain| pyocin protein| pyocin large subunit family protei| pyocin.*cytotoxin	Captures likely pyrocines.

**Table 11:** List of Regex terms part 2 that likely indicate toxicity found in a control set of bacterial proteins

Category	Regex Pattern	Rationale
**Autotransporters and Proteases**	autotransporter serine protease	Autotransporter proteins that may also have toxic enzymatic activity.
**Exo- and Enterotoxins**	exotoxin Spe|exotoxin/[|exotoxin SpeL|enterotoxin/[|enterotoxin II|enterotoxin.*family|enterotoxin.*domain	Captures likely exotoxins and enterotoxins.
**Specific Protein Identifiers**	(WP_241193295.1|WP_233395584.1|WP_230016319.1|WP_032488282.1|WP_019777116.1|WP_006729641.1|WP_123162107.1|WP_138210472.1|WP_233592186.1|WP_134812624.1|WP_041640381.1|WP_051462489.1|WP_012907316.1|WP_015331512.1|WP_016523484.1|WP_013316374.1|WP_009601635.1|NP_463732.1|WP_009599859.1|WP_009291561.1|WP_002517003.1|NP_820136.1|WP_011533690.1|WP_051869004.1|WP_139180491.1|WP_164488466.1|WP_219827103.1|WP_006729641.1|WP_019777116.1|WP_032488282.1|WP_230016319.1|WP_233395584.1|WP_241193295.1)	Manually curated list of known toxin of type IV-related proteins. Manual curation based on distance in embedding-space.
**Specific Toxins Names**	VasX| HicA| TisB| TNT| MazF| DinQ|toxin PIN| PezT| IbsA| ShoB| RTX| ParE2| CcdB| HigB| RelE| DqlB| Doc| RtxA |VasX|toxin YdaT| toxin RelG|PAAR|HipA|TNT| YopE| MARTX|popB|PAAR|TcdB| ETA|toxin Cry1Ac	Identifies abbreviations of toxins.
**Other Specific Toxins**	ricin|RICIN|perforin| latrotoxin-related|toxin co-regulated pilus biosynthesis Q family protein| superantigen-like| delta-endotoxin| antimicrobial peptide LCI| type VI secretion system tip protein| polymorphic toxin| latrotoxin-related| exo-alpha-sialidase| killer protein	Captures other important toxins by name or descriptive patterns that denote known toxic proteins.

## Abbreviations

2D: two dimensional
aac: Amino Acid Composition
APAAC: Amphiphilic Pseudo Amino Acid Composition [60]
BLAST: Basic Local Alignment Search Tool
CI: Confidence Interval, here typically used as the 95% CI implying an interval between 1.96*Standard Error
CSM-Toxin: particular toxin predictor [47]
Embs20: first twenty principle components calculated on embeddings using a PCA
embeddings: fixed-size vectors derived from pre-trained pLMs
Exo-Tox: selected high performance predictor introduced in this work called Embs20/SVC during training
FN: False Negative
FP: False Positive
HMMER: tool for finding sequence homologs and alignments [22]
HMMs: Hidden Markov Models
pI: isoelectric point
kNN: K-Nearest Neighbors
LIPO: signal peptide indicating the combination of Sec/SPII
LIPOTAT: signal peptide indicating the combination Tat/SPII
LR: Logistic Regression
ML: machine learning
MCC: Matthews Correlation Coefficient
MMseqs2: Many-against-Many sequence searching tool for clustering sequences [20]
OMVs: Outer Membrane Vesicles
pLMs: protein Language Model
PC: principle components from a PCA
PCA: principle component analysis
PILIN: signal peptide indicating the combination of Sec/SPIII
ProtT5: particular pLM [54]
pseaac: pseudo amino acid composition
PSI-BLAST: Position-Specific Iterative Basic Local Alignment Search Tool [19]
PSORTb: 3.0b, subcellular localization prediction tool [59]
Regex: regular expression
RF: Random Forest
ROC-AUC: Receiver Operator Curve - Area Under the Curve
SignalP-6.0: tool to predict the presence of signal peptides [66]
Sec: Secretory pathway, a protein transport system
SE: Standard Error
SOM: Supplementary Online Material
SP: Signal Peptide
SPI-III: signal peptidases 1-3
SVC: Support Vector Classifier
T5: architecture choice in pLMs
T1SS: Type I Secretion System
T3SS: Type III Secretion System
T4SS: Type IV Secretion System
T6SS: Type VI Secretion System
TAT: signal peptide indicating the combination of Tat/SPI
Tat: twin arginine translocation system, a protein transport system
TN: True Negative
TP: True Positive
XGB: extreme Gradient Boosting

## Code availability

All code is written in python or R. All code, corresponding documentation and information for non-programmers on how to reproduce the project is available under, 10.5282/ubm/data.576 and 10.5282/ubm/data.665, too.

## References

[1] Speare L, Cecere AG, Guckes KR, Smith S, Wollenberg MS, Mandel MJ, et al. Bacterial symbionts use a type VI secretion system to eliminate competitors in their natural host. Proc Natl Acad Sci USA. 2018;115(36):E8528–37. 10.1073/PNAS.1808302115. 30127013 10.1073/pnas.1808302115 PMC6130350

[2] Sana TG, Flaugnatti N, Lugo KA, Lam LH, Jacobson A, Baylot V, et al. Salmonella Typhimurium utilizes a T6SS-mediated antibacterial weapon to establish in the host gut. Proc Natl Acad Sci U S A. 2016;113(34):E5044–51. 10.1073/PNAS.1608858113. 27503894 10.1073/pnas.1608858113 PMC5003274

[3] Carbonetti NH, Artamonova GV, Rooijen NV, Ayala VI. Pertussis Toxin Targets Airway Macrophages To Promote Bordetella pertussis Infection of the Respiratory Tract. Infect Immun. 2007;75(4):1713–20. 10.1128/IAI.01578-06. 17242062 10.1128/IAI.01578-06 PMC1865687

[4] Kumar R, Feltrup TM, Kukreja RV, Patel KB, Cai S, Singh BR. toxins Evolutionary Features in the Structure and Function of Bacterial Toxins. Toxins. 2019;11:15. 10.3390/toxins11010015. 30609803 10.3390/toxins11010015 PMC6356308

[5] Brouwer S, Barnett TC, Ly D, Kasper KJ, De Oliveira DMP, Rivera-Hernandez T, et al. Prophage exotoxins enhance colonization fitness in epidemic scarlet fever-causing Streptococcus pyogenes. Nat Commun. 2020;11(1):1–11. 10.1038/s41467-020-18700-5. 33024089 10.1038/s41467-020-18700-5 PMC7538557

[6] Rietschel ET, Brade H, Holst O, Brade L, Müller-Loennies S, Mamat U, et al. Bacterial Endotoxin: Chemical Constitution, Biological Recognition, Host Response, and Immunological Detoxification. In: Current Topics in Microbiology and Immunology. vol. 216. Curr Top Microbiol Immunol; 1996. pp. 39–81. 10.1007/978-3-642-80186-0_3. 10.1007/978-3-642-80186-0_3 8791735

[7] D’Onofrio C, Paradisi F. The influence of bacterial exotoxins and endotoxins on the phagocytic activity of human macrophages in culture. Infection. 1983;11(3):137–43. 10.1007/BF01641292. 6350191 10.1007/BF01641292

[8] Sheehan JR, Sadlier C, O’Brien B. Bacterial endotoxins and exotoxins in intensive care medicine. BJA Educ. 2022;22(6):224–30. 10.1016/J.BJAE.2022.01.003. 35614909 10.1016/j.bjae.2022.01.003 PMC9125418

[9] Roux E, Yersin A. Contribution de l’étude de la diphtérie. Inst. Pasteur. 1888.

[10] Suez J, Zmora N, Segal E, Elinav E. The pros, cons, and many unknowns of probiotics. Nat Med. 2019;25(5):716–29. 10.1038/s41591-019-0439-x. 31061539 10.1038/s41591-019-0439-x

[11] Lerner A, Matthias T. There Are Many More Cons for Probiotics. Israel Med Assoc J IMAJ. 2020;22(2):131. 32043336

[12] Merenstein D, Pot B, Leyer G, Ouwehand AC, Preidis GA, Elkins CA, et al. Emerging issues in probiotic safety: 2023 perspectives. Gut Microbes. 2023;15(1):1–22. 10.1080/19490976.2023.2185034. 10.1080/19490976.2023.2185034 PMC10026873 36919522

[13] Holvoet T, Joossens M, Wang J, Boelens J, Verhasselt B, Laukens D, et al. Assessment of faecal microbial transfer in irritable bowel syndrome with severe bloating. Gut. 2016;66(5):980–2. 10.1136/gutjnl-2016-312513. 27511198 10.1136/gutjnl-2016-312513 PMC5531219

[14] Malard F, Vekhoff A, Lapusan S, Isnard F, D’incan-Corda E, Rey J, et al. Gut microbiota diversity after autologous fecal microbiota transfer in acute myeloid leukemia patients. Nat Commun. 2021;12(1). 10.1038/s41467-021-23376-6. 10.1038/s41467-021-23376-6 PMC8149453 34035290

[15] Choudhury S, Baker MR, Chatterjee S, Kumar H. Botulinum Toxin: An Update on Pharmacology and Newer Products in Development. Toxins. 2021;13(1). 10.3390/toxins13010058. 10.3390/toxins13010058 PMC7828686 33466571

[16] Poulain B, Molgo J, Popoff MR. Clostridial neurotoxins: From the cellular and molecular mode of action to their therapeutic use. In: The Comprehensive Sourcebook of Bacterial Protein Toxins. 4th ed. Amsterdam: Elsevier; 2015. pp. 287–336.

[17] Peigneur S, Tytgat J. Toxins in drug discovery and pharmacology. Toxins. 2018;10(3):10–3. 10.3390/toxins10030126. 10.3390/toxins10030126 PMC5869414 29547537

[18] Altschul SF, Gish W, Miller W, Myers EW, Lipman DJ. Basic local alignment search tool. J Mol Biol. 1990;215(3):403–10. 10.1016/S0022-2836(05)80360-2. 2231712 10.1016/S0022-2836(05)80360-2

[19] Altschul SF, Madden TL, Schäffer AA, Zhang J, Zhang Z, Miller W, et al. Gapped BLAST and PSI-BLAST: A new generation of protein database search programs. Nucleic Acids Res. 1997;25(17):3389–402. 10.1093/nar/25.17.3389. 9254694 10.1093/nar/25.17.3389 PMC146917

[20] Steinegger M, Söding J. MMseqs2 enables sensitive protein sequence searching for the analysis of massive data sets. Nat Biotechnol. 2017;35(11):1026–8. 10.1038/nbt.3988. 29035372 10.1038/nbt.3988

[21] Krogh A, Brown M, Mian IS, Sjölander K, Haussler D. Hidden Markov Models in Computational Biology: Applications to Protein Modeling. J Mol Biol. 1994;235(5):1501–31. 10.1006/JMBI.1994.1104. 8107089 10.1006/jmbi.1994.1104

[22] Eddy SR. Accelerated Profile HMM Searches. Citation Eddy SR. 2011;7(10):1002195. 10.1371/journal.pcbi.1002195. 10.1371/journal.pcbi.1002195 PMC3197634 22039361

[23] Alley EC, Turpin M, Liu AB, Kulp-McDowall T, Swett J, Edison R, et al. A machine learning toolkit for genetic engineering attribution to facilitate biosecurity. Nat Commun. 2020;11(1):1–12. 10.1038/s41467-020-19612-0. 33293535 10.1038/s41467-020-19612-0 PMC7722865

[24] Pan Y, Wang S, Zhang Q, Lu Q, Su D, Zuo Y, et al. Analysis and prediction of animal toxins by various Chou’s pseudo components and reduced amino acid compositions. J Theor Biol. 2019;462:221–9. 10.1016/J.JTBI.2018.11.010. 30452961 10.1016/j.jtbi.2018.11.010

[25] Naamati G, Askenazi M, Linial M. ClanTox: a classifier of short animal toxins. Nucleic Acids Res. 2009;37:363–8. 10.1093/nar/gkp299. 10.1093/nar/gkp299 PMC2703885 19429697

[26] Cole TJ, Brewer MS, Cole TJ. TOXIFY: A deep learning approach to classify animal venom proteins. PeerJ. 2019;2019(6). 10.7717/peerj.7200. 10.7717/peerj.7200 PMC6601600 31293833

[27] Gacesa R, Barlow DJ, Long PF. Machine learning can differentiate venom toxins from other proteins having non-toxic physiological functions. PeerJ Comput Sci. 2016;2016(10). 10.7717/PEERJ-CS.90.

[28] Gupta S, Kapoor P, Chaudhary K, Gautam A, Kumar R, Raghava GPSS, et al. In Silico Approach for Predicting Toxicity of Peptides and Proteins. PLoS ONE. 2013;8(9):e73957. 10.1371/journal.pone.0073957. 24058508 10.1371/journal.pone.0073957 PMC3772798

[29] Wei L, Ye X, Xue Y, Sakurai T, Wei L. ATSE: a peptide toxicity predictor by exploiting structural and evolutionary information based on graph neural network and attention mechanism. Brief Bioinforma. 2021;22(5). 10.1093/BIB/BBAB041. 10.1093/bib/bbab041 33822870

[30] Guan J, Xie P, Meng D, Yao L, Yu D, Chiang YC, et al. ToxiPep: Peptide toxicity prediction via fusion of context-aware representation and atomic-level graph. Comput Struct Biotechnol J. 2025;27:2347–58. 10.1016/j.csbj.2025.05.039. 40529180 10.1016/j.csbj.2025.05.039 PMC12171765

[31] Yu Q, Zhang Z, Liu G, Li W, Tang Y. ToxGIN: an In silico prediction model for peptide toxicity via graph isomorphism networks integrating peptide sequence and structure information. Brief Bioinforma. 2024;25(6). 10.1093/bib/bbae583. 10.1093/bib/bbae583 PMC11555482 39530430

[32] Wei L, Ye X, Sakurai T, Mu Z, Wei L. ToxIBTL: Prediction of peptide toxicity based on information bottleneck and transfer learning. Bioinformatics. 2022;38(6):1514–24. 10.1093/bioinformatics/btac006. 34999757 10.1093/bioinformatics/btac006

[33] Rathore AS, Choudhury S, Arora A, Tijare P, Raghava GPS. ToxinPred 3.0: An improved method for predicting the toxicity of peptides. vol. 179. Elsevier; 2024. 10.1016/j.compbiomed.2024.108926. 10.1016/j.compbiomed.2024.108926 39038391

[34] Agüero-Chapin G, Pérez-Machado G, Molina-Ruiz R, Pérez-Castillo Y, Morales-Helguera A, Vasconcelos V, et al. TI2BioP: Topological Indices to BioPolymers. Its practical use to unravel cryptic bacteriocin-like domains. Amino Acids. 2011;40(2):431–42. 10.1007/s00726-010-0653-9. 20563611 10.1007/s00726-010-0653-9

[35] Akhter S, Miller JH. BPAGS: a web application for bacteriocin prediction via feature evaluation using alternating decision tree, genetic algorithm, and linear support vector classifier. Front Bioinforma. 2023;3(January):1–11. 10.3389/fbinf.2023.1284705. 10.3389/fbinf.2023.1284705 PMC10807691 38268970

[36] Akhter S, Miller JH. BaPreS: a software tool for predicting bacteriocins using an optimal set of features. BMC Bioinformatics. 2023;24(1):1–14. 10.1186/s12859-023-05330-z. 37592230 10.1186/s12859-023-05330-z PMC10433575

[37] Gao M, Song C, Liu T. PLM-T3SE: Accurate Prediction of Type III Secretion Effectors Using Protein Language Model Embeddings. J Cell Biochem. 2024;126(1). 10.1002/jcb.30642. 10.1002/jcb.30642 39164870

[38] Jing R, Wen T, Liao C, Xue L, Liu F, Yu L, et al. DeepT3 2.0: improving type III secreted effector predictions by an integrative deep learning framework. NAR Genomics Bioinforma. 2021;3(4):1–14. 10.1093/nargab/lqab086. 10.1093/nargab/lqab086 PMC8489581 34617013

[39] Saha S, Raghava GP. BTXpred: prediction of bacterial toxins. In Silico Biol. 2007;7(4-5):405-12. PMID: 18391233. 18391233

[40] Ahn SY, Kim M, Bae JE, Bang IS, Lee SW. Reliability of the In Silico Prediction Approach to In Vitro Evaluation of Bacterial Toxicity. Sensors. 2022;22(17). 10.3390/s22176557. 10.3390/s22176557 PMC9459819 36081016

[41] de Nies L, Lopes S, Busi SB, Galata V, Heintz-Buschart A, Laczny CC, et al. PathoFact: a pipeline for the prediction of virulence factors and antimicrobial resistance genes in metagenomic data. Microbiome. 2021;9(1):49. 10.1186/s40168-020-00993-9. 33597026 10.1186/s40168-020-00993-9 PMC7890817

[42] Li G, Zhou J, Luo J, Liang C. Accurate prediction of virulence factors using pre-train protein language model and ensemble learning. BMC Genomics. 2025;26(1). 10.1186/s12864-025-11694-8. 10.1186/s12864-025-11694-8 PMC12093764 40399812

[43] Rentzsch R, Deneke C, Nitsche A, Renard BY. Predicting bacterial virulence factors - evaluation of machine learning and negative data strategies. Brief Bioinforma. 2020;21(5):1596–608. 10.1093/bib/bbz076. 10.1093/bib/bbz076 32978619

[44] Liu Y, Cao X, Li J, Li T, Li J, Ma X, et al. Advancing virulence factor prediction using protein language model. Res Cube. 2024. 10.21203/rs.3.rs-4664562/v1. 10.1186/s12915-025-02374-w PMC12522538 41088209

[45] Chen C, Xu Y, Ouyang J, Xiong X, Łabaj PP, Chmielarczyk A, et al. VirulentHunter: deep learning-based virulence factor predictor illuminates pathogenicity in diverse microbial contexts. Brief Bioinforma. 2025;26(3). 10.1093/bib/bbaf271. 10.1093/bib/bbaf271 PMC12167765 40518950

[46] Caceres-Delpiano J, Ibañez R, Correa S, Dunne MP, Retamal P, Álvarez L, et al. Deep learning model for the prediction and classification of protein toxins across all domains of life. bioRxiv. 2021. 10.1101/2021.06.29.450401.

[47] Morozov V, Rodrigues CHM, Ascher DB. CSM-Toxin: A Web-Server for Predicting Protein Toxicity. Pharmaceutics. 2023;15(2):431. 10.3390/pharmaceutics15020431. 36839752 10.3390/pharmaceutics15020431 PMC9966851

[48] Beltrán JF, Herrera-Belén L, Parraguez-Contreras F, Farías JG, Machuca-Sepúlveda J, Short S. MultiToxPred 1.0: a novel comprehensive tool for predicting 27 classes of protein toxins using an ensemble machine learning approach. BMC Bioinformatics. 2024;25(1):1–12. 10.1186/s12859-024-05748-z. 38609877 10.1186/s12859-024-05748-z PMC11010298

[49] Zhu L, Fang Y, Liu S, Shen HB, De Neve W, Pan X. ToxDL 2.0: Protein toxicity prediction using a pretrained language model and graph neural networks. Comput Struct Biotechnol J. 2025;27(April):1538–49. 10.1016/j.csbj.2025.04.002. 40276117 10.1016/j.csbj.2025.04.002 PMC12018212

[50] Brandes N, Ofer D, Peleg Y, Rappoport N, Linial M. ProteinBERT: a universal deep-learning model of protein sequence and function. Bioinformatics. 2022;38(8):2102–10. 10.1093/BIOINFORMATICS/BTAC020. 35020807 10.1093/bioinformatics/btac020 PMC9386727

[51] Villegas-Morcillo A, Makrodimitris S, Van Ham RCHJ, Gomez AM, Sanchez V, Reinders MJT. Unsupervised protein embeddings outperform hand-crafted sequence and structure features at predicting molecular function. Bioinformatics. 2021;37(2):162–70. 10.1093/bioinformatics/btaa701. 32797179 10.1093/bioinformatics/btaa701 PMC8055213

[52] Heinzinger M, Elnaggar A, Wang Y, Dallago C, Nechaev D, Matthes F, et al. Modeling aspects of the language of life through transfer-learning protein sequences. BMC Bioinformatics. 2019;20(1):1–17. 10.1186/s12859-019-3220-8. 31847804 10.1186/s12859-019-3220-8 PMC6918593

[53] Rives A, Meier J, Sercu T, Goyal S, Lin Z, Liu J, et al. Biological structure and function emerge from scaling unsupervised learning to 250 million protein sequences. Proc Natl Acad Sci USA. 2021;118(15). 10.1073/pnas.2016239118. 10.1073/pnas.2016239118 PMC8053943 33876751

[54] Elnaggar A, Heinzinger M, Dallago C, Rehawi G, Wang Y, Jones L, et al. ProtTrans: Toward Understanding the Language of Life Through Self-Supervised Learning. IEEE Trans Pattern Anal Mach Intell. 2022;44(10):7112–27. 10.1109/TPAMI.2021.3095381. 34232869 10.1109/TPAMI.2021.3095381

[55] Erckert K, Rost B. Assessing the role of evolutionary information for enhancing protein language model embeddings. Sci Rep. 2024;14(1):1–14. 10.1038/s41598-024-71783-8. 39237735 10.1038/s41598-024-71783-8 PMC11377704

[56] Krüger T, Koludarov I, Littmann M, Rost B, Jimenez-Soto LF. Toxin Data Quality: A Critical Examination of Bacterial Exotoxins and Animal Toxins. Preprint. 2024. 10.21203/RS.3.RS-4376594/V1. 10.1186/s13104-025-07438-2 PMC12492933 41039617

[57] Bairoch A, Apweiler R. The SWISS-PROT protein sequence data bank and its new supplement TREMBL. Nucleic Acids Res. 1996;24(1):21–5. 10.1093/NAR/24.1.21. 8594581 10.1093/nar/24.1.21 PMC145613

[58] Sayers EW, Bolton EE, Brister JR, Canese K, Chan J, Comeau DC, et al. Database resources of the national center for biotechnology information. Nucleic Acids Res. 2022;50(D1):D20–6. 10.1093/NAR/GKAB1112. 34850941 10.1093/nar/gkab1112 PMC8728269

[59] Yu NY, Wagner JR, Laird MR, Melli G, Rey S, Lo R, et al. PSORTb 3.0: improved protein subcellular localization prediction with refined localization subcategories and predictive capabilities for all prokaryotes. Bioinformatics (Oxford, England). 2010;26(13):1608–15. 10.1093/BIOINFORMATICS/BTQ249. 20472543 10.1093/bioinformatics/btq249 PMC2887053

[60] Chou KC. Using amphiphilic pseudo amino acid composition to predict enzyme subfamily classes. Bioinformatics (Oxford, England). 2005;21(1):10–9. 10.1093/bioinformatics/bth466. 15308540 10.1093/bioinformatics/bth466

[61] Chen Z, Liu X, Zhao P, Li C, Wang Y, Li F, et al. iFeatureOmega: an integrative platform for engineering, visualization and analysis of features from molecular sequences, structural and ligand data sets. Nucleic Acids Res. 2022;50(W1):W434–47. 10.1093/nar/gkac351. 35524557 10.1093/nar/gkac351 PMC9252729

[62] Chicco D, Tötsch N, Jurman G. The matthews correlation coefficient (Mcc) is more reliable than balanced accuracy, bookmaker informedness, and markedness in two-class confusion matrix evaluation. BioData Min. 2021;14:1–22. 10.1186/s13040-021-00244-z. 33541410 10.1186/s13040-021-00244-z PMC7863449

[63] Boughorbel S, Jarray F, El-Anbari M. Optimal classifier for imbalanced data using Matthews Correlation Coefficient metric. PLoS ONE. 2017;12(6):1–17. 10.1371/journal.pone.0177678. 10.1371/journal.pone.0177678 PMC5456046 28574989

[64] National Center for Biotechnology Information. BLAST® Command Line Applications User Manual. 2008. https://www.ncbi.nlm.nih.gov/books/NBK279674/. Accessed Oct 2023.

[65] Van Kempen M, Kim SS, Tumescheit C, Mirdita M, Lee J, Gilchrist CLM, et al. Nature biotechnology Fast and accurate protein structure search with Foldseek. Nat Biotechnol. 2024;42:243–6. 10.1038/s41587-023-01773-0. 37156916 10.1038/s41587-023-01773-0 PMC10869269

[66] Teufel F, Almagro Armenteros JJ, Johansen AR, Gíslason MH, Pihl SI, Tsirigos KD, et al. SignalP 6.0 predicts all five types of signal peptides using protein language models. Nat Biotechnol. 2022;40(7):1023–5. 10.1038/s41587-021-01156-3. 34980915 10.1038/s41587-021-01156-3 PMC9287161

[67] Cantu VA, Salamon P, Seguritan V, Redfield J, Salamon D, Edwards RA, et al. PhANNs, a fast and accurate tool and web server to classify phage structural proteins. PLOS Comput Biol. 2020;16(11):e1007845. 10.1371/JOURNAL.PCBI.1007845. 33137102 10.1371/journal.pcbi.1007845 PMC7660903

[68] Russell DA, Hatfull GF. PhagesDB: The actinobacteriophage database. Bioinformatics. 2017;33(5):784–6. 10.1093/bioinformatics/btw711. 28365761 10.1093/bioinformatics/btw711 PMC5860397

[69] Zhang L, Zhang C, Gao R, Yang R. An ensemble method to distinguish bacteriophage virion from non-virion proteins based on protein sequence characteristics. Int J Mol Sci. 2015;16(9):21734–58. 10.3390/ijms160921734. 26370987 10.3390/ijms160921734 PMC4613277

[70] Saha SRG. Prediction of neurotoxins based on their function and source. In Silico Biol. 2007;7(4–5):369–87. 18391230

[71] Sharma N, Naorem LD, Jain S, Raghava GP. ToxinPred2: An improved method for predicting toxicity of proteins. Brief Bioinforma. 2022;23(5):1–12. 10.1093/bib/bbac174. 10.1093/bib/bbac174 35595541

[72] Datta D, Muthiah S, Butler P, Islam MR, Warren A, Ramakrishnan N. ProtTox: Toxin identification from Protein Sequences. bioRxiv. 2020;2020.04.18.048439. 10.1101/2020.04.18.048439.

[73] Zhao Z, Gui J, Yao A, Le NQK, Chua MCH. Improved Prediction Model of Protein and Peptide Toxicity by Integrating Channel Attention into a Convolutional Neural Network and Gated Recurrent Units. ACS Omega. 2022;7(44):40569–77. 10.1021/acsomega.2c05881. 36385847 10.1021/acsomega.2c05881 PMC9647964

[74] Pan X, Zuallaert J, Wang X, Shen HB, Campos EP, Marushchak DO, et al. ToxDL: Deep learning using primary structure and domain embeddings for assessing protein toxicity. Bioinformatics. 2020;36(21):5159–68. 10.1093/bioinformatics/btaa656. 10.1093/bioinformatics/btaa656 32692832

[75] Weissenow K, Heinzinger M, Rost B. Protein language-model embeddings for fast, accurate, and alignment-free protein structure prediction. Structure (London, England: 1993). 2022;30(8):1169-1177.e4. 35609601 10.1016/j.str.2022.05.001

[76] Villegas-Morcillo A, Gomez AM, Sanchez V. An analysis of protein language model embeddings for fold prediction. Brief Bioinforma. 2022;23(3):bbac142. 10.1093/bib/bbac142. 10.1093/bib/bbac142 35443054

[77] de Lucrezia D, Slanzi D, Poli I, Polticelli F, Minervini G. Do natural proteins differ from random sequences polypeptides? natural vs. random proteins classification using an evolutionary neural network. PLoS ONE. 2012;7(5). 10.1371/journal.pone.0036634. 10.1371/journal.pone.0036634 PMC3353917 22615786

[78] Yu JF, Cao Z, Yang Y, Wang CL, Su ZD, Zhao YW, et al. Natural protein sequences are more intrinsically disordered than random sequences. Cell Mol Life Sci. 2016;73(15):2949–57. 10.1007/s00018-016-2138-9. 26801222 10.1007/s00018-016-2138-9 PMC4937073

[79] Tsygvintsev A. Natural vs. random protein sequences: the novel neural network approach based on time series analysis. J Protein Proteomics. 2020;11(1):11–6. 10.1007/s42485-020-00029-8.

[80] Naureen Z, Dautaj A, Anpilogov K, Camilleri G, Dhuli K, Tanzi B, et al. Bacteriophages presence in nature and their role in the natural selection of bacterial populations. Acta Bio-Med Atenei Parmensis. 2020;91(13–S):e2020024. 10.23750/abm.v91i13-S.10819. 10.23750/abm.v91i13-S.10819 PMC8023132 33170167

[81] Jamet A, Touchon M, Ribeiro-Gonçalves B, Carriço JA, Charbit A, Nassif X, et al. A widespread family of polymorphic toxins encoded by temperate phages. BMC Biol. 2017;15(1):75. 10.1186/s12915-017-0415-1. 28851366 10.1186/s12915-017-0415-1 PMC5576092

[82] Boyd EF, Brüssow H. Common themes among bacteriophage-encoded virulence factors and diversity among the bacteriophages involved. Trends Microbiol. 2002;10(11):521–9. 10.1016/S0966-842X(02)02459-9. 12419617 10.1016/s0966-842x(02)02459-9

[83] Ektefaie Y, Shen A, Bykova D, Marin M, Zitnik M, Farhat M. Evaluating generalizability of artificial intelligence models for molecular datasets. bioRxiv. 2024;2024.02.25.581982. 10.1101/2024.02.25.581982.

[84] Moshiri M, Hamid F, Etemad L. Ricin Toxicity: Clinical and Molecular Aspects. Rep Biochem Mol Biol. 2016;4(2):60–5. 27536698 PMC4986263

[85] Soliman SSM, Baldin C, Gu Y, Singh S, Gebremariam T, Swidergall M, et al. Mucoricin is a ricin-like toxin that is critical for the pathogenesis of mucormycosis. Nat Microbiol. 2021;6(3):313–26. 10.1038/s41564-020-00837-0. 33462434 10.1038/s41564-020-00837-0 PMC7914224

